# Beyond prion‐like spreading in neurodegenerative disease

**DOI:** 10.1002/alz.70789

**Published:** 2025-10-21

**Authors:** Georg Meisl, James B. Rowe, David Klenerman

**Affiliations:** ^1^ Yusuf Hamied Department of Chemistry University of Cambridge Cambridge UK; ^2^ Dementia Research Institute University of Cambridge Cambridge UK; ^3^ Department of Clinical Neuroscience and Cambridge University Hospitals NHS Trust University of Cambridge Cambridge UK

**Keywords:** aggregate removal, disease mechanisms, neurodegeneration, prion‐like, protein aggregation, protein homeostasis

## Abstract

To design effective therapies for neurodegenerative diseases, it is critical to understand the processes that trigger protein aggregation in sequential brain regions as the disease progresses. Aggregates formed in many neurodegenerative diseases, including Alzheimer's and Parkinson's disease, are capable of seeding, leading to the proposal to regard them all as prion‐like. We here argue that the utility of this classification is limited; the terms *protein misfolding* and *aggregation‐related diseases* describe the general class of diseases, and the connotation of *prion‐like* that the spreading of infectious prions is the rate‐limiting process narrows the view of possible mechanisms. Instead, we suggest four factors along which to compare different diseases and model systems, providing a clearer basis to consider the different ways in which pathology can spread, account for factors beyond the aggregating protein, such as declining protein homeostasis with age, and understand the differences between model systems and human disease.

**Highlights:**

- Four aspects by which to classify neurodegenerative diseases are proposed.
- Aggregates in health and inflammation are important factors.
- Prion‐like spreading classification is not sufficient to capture the necessary nuance.
- Different diseases and model systems are dominated by different aspects.

## 1 DIMENSIONS TO CLASSIFY DISEASES

A wide range of disease is linked to the misfolding and aggregation of proteins.^1^, ^2^Most proteins that aggregate in neurodegenerative disease can auto‐catalytically trigger the formation of more aggregates, at least in well‐controlled in vitro conditions^3^. This auto‐catalysis is considered a quintessential prion‐like property, but prions are not special in this regard, and the self‐replication of ordered, assembled structures is a common physical phenomenon that occurs, for example, even in the formation of inorganic crystals.^4^, ^5^ While protein systems are more complex, due to the multitude of conformations that an individual protein can adopt, many features appear in the much simpler inorganic systems, such as significantly increased rates in seeded systems and the faithful propagation of strains, or conformations. Accordingly, it is not surprising that self‐replicating aggregates are observed across model systems of neurodegenerative disease and that “disease” in these model systems can be induced by the introduction of preformed aggregates or seeds.^6^, ^7^, ^8^, ^9^ However, while the intrinsic ability of aggregates to self‐replicate is common across disease‐associated proteins, the crucial question is to what degree it drives the progressive appearance of pathology in brain regions, in particular for the many, sporadic neurodegenerative diseases that do not involve the aggregation of a prion protein. These include Parkinson's disease (PD) and Alzheimer's disease (AD) but also less common tauopathies, such as progressive supranuclear palsy, corticobasal degeneration, or frontotemporal dementia, as well as other synucleinopathies and aggregation‐related diseases. Multiple other processes, such as inflammation, decline in protein homeostasis, and aging,^10^, ^11^, ^12^, ^13^ have been highlighted as important factors, not just as downstream effects of protein aggregation, but also in the emergence of disease. The overemphasis on prion‐like seeding descriptions of disease is at odds with these key observations^14^.

**FIGURE 1:**
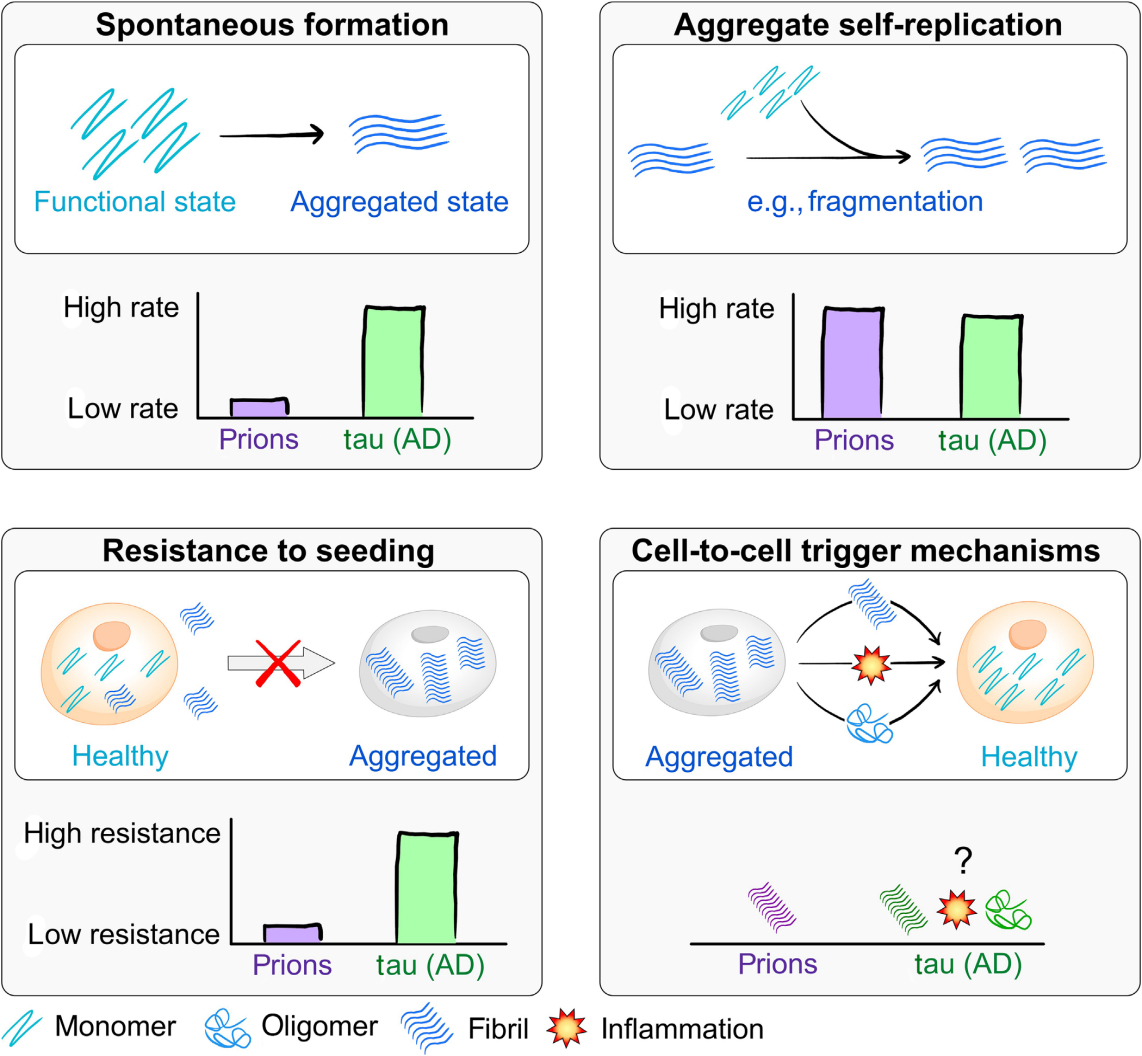
Four aspects on which to classify aggregation‐related diseases and examine the similarity to prion disease. Schematics of each aspect are presented, and the example of prion disease and tau in AD is given to illustrate them. *Spontaneous formation* describes the process by which new aggregates form without the involvement of existing ones. The rate of this process appears to be much higher for tau than for the prion protein PrP, resulting in the widespread appearance of tau aggregates in human brains of both healthy and diseased individuals. *Aggregate self‐replication* denotes the intrinsic ability of existing aggregates to catalyze the formation of new ones, for example, by fragmentation of secondary nucleation in vitro. The rate of this process is high for both proteins. *Resistance to seeding* is the ability of cells to cope with exposure to aggregated proteins, without entering a runaway aggregation state and producing additional aggregates. Again, cells seem to be more able to cope with tau aggregates than prions. Finally, *cell‐to‐cell triggers* are the processes by which one cell triggers aggregation in another. In prion disease, this is likely exclusively by transfer of a prion from one cell to another. For tau in Alzheimer's disease, less direct mechanisms, such as the release of stressors of the protein homeostasis system, may be responsible for the triggering of aggregation.

We propose that, rather than focusing on a “prion‐like” model, other aspects of aggregation behavior should be used to delineate different situations and diseases. These provide a clearer way to classify disease, along dimensions that are less ambiguous and reduce the potential for misinterpretation. They are illustrated in Figure 1, with classical prion disease and tauopathy in AD as qualitative examples for how different diseases require different classifications.

In addition to the dynamics of auto‐catalysis, we identify four critical aspects that govern the appearance of protein aggregates:

- *Aggregate self‐replication*, the ability of existing aggregates to trigger the formation of more aggregates; while this is often termed *prion‐like replication*, this term provides no utility over the clearly defined term *self‐replication* and carries a significantly increased risk of misinterpretation;
- *Spontaneous or de novo aggregate formation* without involvement of existing aggregates;
- *Resistance to seeding*, the ability of a cell to tolerate the presence of aggregates without entering a runaway aggregation state;
- *Cell‐to‐cell trigger mechanisms* by which cells with aggregates trigger aggregation in other cells.

Where possible, we aim to avoid terms that may be misleading without adding further clarification, such as *spreading*, which can imply the transfer of aggregates or simply the increased spatial extent of pathology, and *propagation*, which can be used to mean the self‐replication of an aggregate or the progression of pathology through space. While self‐replication and spontaneous formation are relevant processes across aggregating proteins, the final two aspects concern the formation of predominantly intracellular aggregates, such as tau or α‐synuclein. Considering these four aspects allows a more nuanced discussion of the drivers of disease.

## 2 THE CLASSIC PRION CASE

In classical prion disease, spontaneous formation is a rare event, but resistance to subsequent seeding is very low, and cell‐to‐cell spread occurs by transfer of self‐replication‐competent aggregates. With this mechanism, a basic prion‐like process would proceed along the following lines. (1) Initially, a fibrillar aggregate forms in one neuron in one region of the brain, as a low‐probability, spontaneous event. (2) This aggregate self‐replicates into new aggregates within a cell. (3) Some of these fibrils enter neighboring cells, easily triggering an aggregation cascade there. (4) These seeding and transfer processes lead to an increasing spatial extent of aggregates across the brain, with new aggregates having the same structure as the initial fibrillar aggregate.

## 3 LOW BARRIERS TO SPONTANEOUS AGGREGATE FORMATION

In contrast to classical prion disease, the initial spontaneous formation of aggregates may be much more common in other neurodegenerative diseases. Detectable levels of tau seeds have been found even in the early stages of AD and in areas for which traditional tangles‐based staging suggests pathology only in the last stage of disease (e.g., primary visual cortex).^15^ It is difficult to reconcile the high levels of seeds in early stages of disease with a single seed formation event. Rather, multiple independent spontaneous formation events in the same brain are likely to have taken place.^16^ Supporting these observations, induced pluripotent stem cell neurons in cell culture are found to produce aggregates at a high rate, which increases in the presence of stressors.^17^

Note that other observations, such as the presence of disease‐specific folds or *strains*,^18^ sometimes used as an argument that a single fibril serves as the source of all other fibrils, does not rule out more common spontaneous aggregation events: It is well documented in vitro that different conditions and small modifications to the proteins affect fibrillar polymorphs and replication rates, again, much like in the context of inorganic crystal formation. Therefore, conditions in a patient's brain could predispose the protein to adopt a specific aggregated fold without having originated from a single seed.

## 4 AGGREGATE REMOVAL AND RESISTANCE TO SEEDING

Where spontaneous aggregate formation is common, the organism will not be able to simply rely on the vanishingly low probability of initial seed formation to prevent pathology, as in classical prion disease. Instead, resistance to the effects of seed self‐replication, such as aggregate removal, may play a more important role in keeping the system from runaway aggregation. When aggregate removal processes are present, runaway aggregation within a cell is no longer necessarily triggered by the appearance of the first seed.^19^ Instead, aggregates are formed and removed frequently, leading to a low population of aggregates even in healthy cells. The switch to runaway aggregation and disease occurs when removal no longer keeps up with aggregate production.^20^ We refer to this as *homeostasis disruption*.

Super‐resolution imaging of tau aggregates from the frontal cortex confirms aggregates are present even in healthy controls and only found small differences between the aggregates found in AD and in control brains.^21^ Similarly, α‐synuclein aggregates were detected in both PD and control brains.^22^, ^23^ This indicates that for these proteins, aggregated species are common even in health, supporting the importance of removal mechanisms in keeping aggregation in check. Evidence for aggregate removal and resistance to seeding also exists in model systems, such as the resistance of neuronal slice cultures to tau seeding.^7^ Together, these data of AD and PD paint a picture of aggregate formation being a relatively common event, but aggregate removal processes preventing runaway aggregation, a clear contrast to classical prion diseases.

## 5 SPATIAL SPREADING MECHANISMS

When the key prerequisite to trigger pathological changes in a cell is homeostasis disruption, indirect mechanisms for the spread of pathology can become important: Species other than self‐replicating aggregates may be involved in determining the increasing spatial extent of pathology over time, and progression may not require the spread of pathological seeds. By analogy, one can think of a beach getting bigger as the tide goes out: Although its spatial extent increases, this is not due to a spreading of sand. Similarly, the increasing extent of pathology does not necessitate a movement of seeds. For example, fibrils or small non‐fibrillar aggregates could induce homeostasis disruption and thereby promote protein aggregation indirectly. Alternatively, aggregate‐induced inflammation of glia can lead to the production of proinflammatory cytokines, which may in turn induce homeostasis disruption in nearby cells and brain regions.^17^ While these mechanisms do not involve the direct transfer of aggregates to induce seeding, they are still auto‐catalytic, that is, cells that are already in a runaway aggregation state trigger the aggregation in other healthy cells. Finally, the fact that even in advanced disease there are significant numbers of non‐fibrillar aggregates is at odds with a fibril‐only model and requires that any realistic model must allow for the presence of such species.

## 6 MODEL SYSTEMS AND CHANGES IN DOMINANT MECHANISMS

When multiple processes can play a role, one needs to consider that the dominant process driving overall disease progression may change between different forms of the human disease and between model systems. For example, even in systems where spontaneous formation is a relatively common event, the addition of enough preformed seeds will result in behavior dominated by these seeds rather than spontaneous formation: In non‐physiological states such as dural transplants in humans or homogenate transfection in preclinical models,^24^, ^25^ massive numbers of seeds are introduced to the system. This switches the disease dynamics to being dominated by seeds triggering runaway aggregation. However, the dominance of seeding as a trigger in this scenario does not imply that the natural history of the aggregation‐related disease is governed by the same rate‐limiting process as in these rare cases. There may be other upstream points that could be used in therapeutic interventions to prevent the triggering of natural disease but that are no longer useful in the high‐seed special case. For example, a rebalancing of aggregate production and removal may stabilize a decline that would trigger AD, but the same strategy may not be effective when massive amounts of seeds are introduced.

This fact fundamentally complicates the investigation of the mechanism by which sporadic human disease arises: In order to observe the progression on reasonable timescales in model systems, specific processes have to be accelerated, inevitably shifting the dominance of the system. Great care must therefore be taken when extrapolating findings on dominant mechanisms from model systems to human disease.

## 7 CONCLUSION

In conclusion, there is little doubt that the formation of protein aggregates plays a central role in these diseases^1^, ^2^, ^26^ and that these aggregates can self‐replicate in the right circumstances.^3^ Yet, this does not imply that all neurodegenerative diseases with protein aggregation should be classified as prion‐like. We urge an alternative approach that considers the different factors in each disease and situation when comparing and contrasting aggregation‐related diseases. This includes taking into account the potential importance of cell‐autonomous processes and of removal mechanisms and considering species other than fibrillar aggregates as drivers of disease progression. It is essential to consider these alternative models and test them quantitatively if the right therapeutic strategies are to be pursued. The importance of using human data, rather than data from model systems, as ultimate verification of proposed mechanisms cannot be overstated. Additional data, in particular on inflammation and protein homeostatic processes, in human brains, across stages of disease, are urgently needed to establish which mechanisms of initiation and progression are viable therapeutic targets in different neurodegenerative diseases.

## AUTHOR CONTRIBUTIONS

All authors were involved in the conception of the study and the writing of the paper. All authors read and approved the final manuscript.

## CONFLICT OF INTEREST STATEMENT

None related to the current work. G.M. is a consultant for WaveBreak Therapeutics. J.B.R. has undertaken consultancy for Astronautx, Alector, Astex, Asceneuron, Curasen, CumulusNeuro, ClinicalInk, Eisai, Ferrer, Prevail, ICG, and SVHealth. D.K. declares no conflicts of interest. Author disclosures are available in the Supporting Information.

## Supporting information



Supporting Information
